# A Qualitative Study of Clinician Barriers and Facilitators to De-escalation of Thyroid Stimulating Hormone Suppression in Thyroid Cancer Survivors

**DOI:** 10.1016/j.eprac.2025.12.009

**Published:** 2025-12-17

**Authors:** Nina Francis-Levin, Chiu Yi Tan, Brittany L. Gay, Megan R. Haymart, Maria Papaleontiou

**Affiliations:** 1 Division of Metabolism, Endocrinology and Diabetes, Department of Internal Medicine, and Institute of Gerontology, University of Michigan, Ann Arbor, Michigan; 2 School of Social Policy & Practice, University of Pennsylvania, Philadelphia, Pennsylvania; 3 Division of Metabolism, Endocrinology and Diabetes, Department of Internal Medicine, University of Michigan, Ann Arbor, Michigan

**Keywords:** de-escalation, survivorship, thyroid cancer, thyroid hormone, TSH suppression

## Abstract

**Objective:**

Most patients with differentiated thyroid cancer have low-risk disease and excellent prognosis. Thyroid stimulating hormone (TSH) suppression therapy after initial treatment may be unnecessary and potentially harmful for survivors with recurrence-free, low- or intermediate-risk thyroid cancer. Little is known about clinician-reported barriers and facilitators to reducing thyroid cancer survivors’ thyroid hormone dose with the goal of aiming for TSH in the normal reference range.

**Methods:**

Clinicians from the fields of endocrinology (*n* = 8) and primary care (*n* = 7) were recruited through convenience/snowball sampling to participate in semistructured focus groups. Data collection and analyses were informed by the Theoretical Domains Framework for behavior change, a valuable integrative framework which can facilitate comprehensive assessment of behavioral determinants in qualitative studies. Deductive coding and inductive thematic analysis were conducted.

**Results:**

Participants were majority female (73%) and averaged 14 years in clinical practice (range, 1-22). Barriers and facilitators emerged at the patient-level, clinician-level, and system-level. Key clinician-reported barriers included patient distress/anxiety and misinformation, unclear shared patient survivorship goals and plans between specialties, and clinic visit time constraints. Clinician-reported facilitators included building a trusting relationship, delivery of patient-centered education, and communication and collaboration between specialties to establish shared long-term survivorship plans.

**Conclusions:**

We identified barriers and facilitators to de-escalating TSH suppression therapy in thyroid cancer survivors at multiple levels. Understanding these factors will enable clinicians to provide high-value, patient-centered care in order to reduce overtreatment, patient harm and improve quality of life in thyroid cancer survivors.

## Introduction

Most patients with differentiated thyroid cancer have low-risk disease and excellent prognosis.^1^ The American Thyroid Association (ATA) risk stratification for differentiated thyroid cancer categorizes patients into low-, intermediate-, or high-risk groups based on clinical and pathological features that predict the likelihood of disease recurrence.^2,3^ Long-term thyroid stimulating hormone (TSH) suppression therapy, consisting of supraphysiologic doses of thyroid hormone, used to be standard of care following initial surgery with or without radioactive iodine in patients with differentiated thyroid cancer.^4^ In recent years, experts have emphasized the need for de-escalation of TSH suppression therapy, which may be potentially beneficial for patients with advanced differentiated thyroid cancer but is often unnecessary and potentially harmful for patients with recurrence-free low- or recurrence-free intermediate-risk differentiated thyroid cancer.^5-8^ The 2025 guidelines from the ATA and the National Comprehensive Cancer Network advocate for a goal serum TSH in the normal reference range in these patients.^2,9^

Prior studies have shown that patients with low- and intermediate-risk thyroid cancer without recurrence continue to be treated with suppressive doses of thyroid hormone long-term.^10,11^ A physician survey (*N* = 448) found that 80% of physicians were likely or extremely likely to recommend TSH suppression in patients with intermediate-risk, 50% in patients with low-risk, and 30% in patients with very low-risk papillary thyroid cancer, with almost half reporting they would continue TSH suppression therapy for longer than 5 years.^10^ This inappropriate supraphysiologic dosing of thyroid hormone can result in significant cardiovascular and skeletal adverse events, including atrial fibrillation, stroke, increased cardiovascular mortality, osteoporosis and fractures, as well as symptoms, including anxiety, palpitations, and fatigue that may impair quality of life.^12-18^

Despite a recent emphasis towards de-escalating intensive management of low-risk thyroid cancer,^2,19^ there are currently no available data to guide reducing thyroid hormone dose in patients with recurrence-free, low- or intermediate-risk thyroid cancer who are overtreated. To better understand barriers and facilitators to thyroid hormone dose de-escalation for these patients, we conducted a qualitative study among a group of clinicians practicing in endocrinology and primary care.

## Methods

### Study Design

In this qualitative study, we conducted semistructured focus groups with clinicians from the fields of endocrinology and primary care practicing in the United States to understand barriers and facilitators to thyroid hormone dose de-escalation in patients with recurrence-free, low- or intermediate-risk thyroid cancer. The Standards for Reporting Qualitative Research guideline was followed for this study.^20^ The study was exempt by the University of Michigan Institutional Review Board (HUM00263634).

### Participants and Recruitment

Clinicians from endocrinology and primary care were identified by convenience sampling through the authors’ nationwide professional network based on clinical experience in managing thyroid hormone replacement in adult patients with thyroid cancer in the past year. We used purposeful sampling to ensure variability in clinician specialty, practice setting, geographic location, and years in practice.^21^ The study team contacted potential participants by email to explain the study, invite participation in focus groups, and obtain verbal consent. A $50 gift card was offered to all participating clinicians upon study completion. We also utilized snowball sampling by asking consented participants to suggest other potentially interested clinicians. We collected data until thematic saturation was achieved, which was defined as the emergence of no new concepts, shown to be ascertainable with at least 4 focus groups.^22,23^

### Data Collection

Participants were emailed a link to a Qualtrics questionnaire regarding their demographics and clinical practice characteristics; the first page was an informed consent document.

Four focus groups (2 each with clinicians from endocrinology and primary care) were conducted virtually via 2-way video (Zoom platform) using a semistructured protocol between February and May 2025 and lasting up to 90 min. We used a standardized interview guide developed iteratively by consultation with subject matter and methodology experts. The guide comprised 14 open-ended questions prompting participants to reflect on their knowledge, beliefs, practices, and emotions surrounding de-escalating thyroid hormone dose for patients with recurrence-free, low- or intermediate-risk thyroid cancer (as defined by the 2015 ATA guidelines),^3^ focusing on barriers and facilitators. Questions in the interview guide aligned with the Theoretical Domains Framework (TDF), an integrated theoretical model comprising of 14 domains that identifies and targets individual and collective-level influences on behavior change in the context of (de)implementation.^24^

The focus groups were video- and audio-recorded. The Zoom AI Companion feature was utilized to generate a transcript, which was reviewed for accuracy and deidentified prior to analysis (N.F.L.).

### Data Analysis

An initial codebook was developed in alignment with the TDF domains and was iteratively modified to guide deductive analysis. Two authors with expertise in qualitative analysis (N.F.L., C.Y.T.) independently coded the transcripts, then met to compare coding decisions and resolve discrepancies to achieve consensus.^25^ The first author organized coded excerpts into a master spreadsheet, and then performed inductive, thematic analysis to differentiate barriers and facilitators at different levels (patient, clinician, and system). Final themes were discussed and refined with the study team, then aligned with their corresponding TDF domains based upon our interpretation of the core characteristics of each theme. Owing to the exploratory nature of our study, we did not compare subgroups by clinician specialty.

## Results

### Demographic and Clinical Characteristics

Fifteen clinicians participated in this study (8 from endocrinology and 7 from primary care). Table 1 describes the study sample. The majority of clinicians were female (73%) and practiced in academic medical settings (67%). Most participants practiced in the Midwest (47%) and Northeast (33%) US regions, mainly in suburban settings (53%).

### Barriers to De-escalating TSH Suppression

**Fig. 1.**
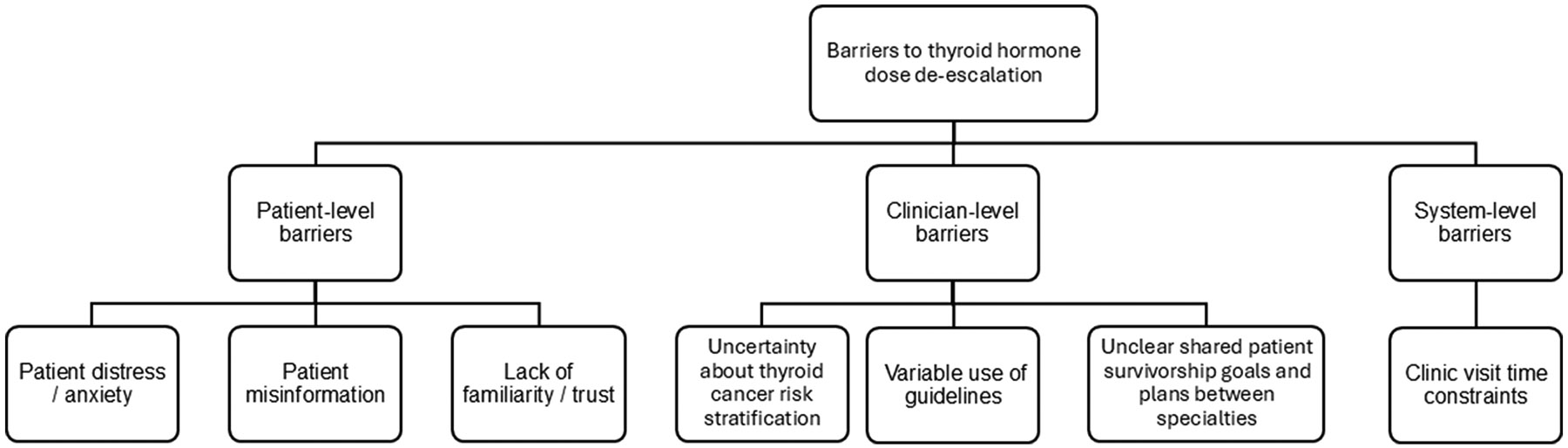
Barriers to de-escalating thyroid hormone dose in patients with thyroid cancer. Figure 1 shows the emergent themes regarding clinician-reported barriers to de-escalating TSH suppression therapy in patients with recurrence-free, low- or intermediate-risk thyroid cancer at the patient, clinician, and system levels. *TSH* = thyroid stimulating hormone.

Figure 1 demonstrates the clinician-reported barriers to de-escalating thyroid hormone dose in patients with recurrence-free, low- and intermediate-risk thyroid cancer. Sample quotations that highlight themes and subthemes and how they map onto the relevant TDF domains are shown in Table 2.

### Patient-Level Barriers

#### Patient Distress/Anxiety

Participants identified patient anxiety as a salient factor when attempting to de-escalate TSH suppression therapy. Clinicians described that patients were anxious about thyroid hormone dose de-escalation potential side effects such as “weight gain, hair loss, and fatigue,” especially among those on TSH suppression therapy for an extensive time period. Clinicians described that patient distress stemmed from the notion that an unsuppressed TSH would precipitate thyroid cancer recurrence, with the term “cancer” adding “another layer to patient anxiety” (PCP number 6). Participants noted that long-term adverse effects from TSH suppression, such as atrial fibrillation or osteoporosis, did not necessarily precipitate additional patient anxiety. Clinicians struggled to convince patients without symptoms of the “clinical consequences of staying over-suppressed” (PCP number 6) when the current risk of long-term adverse effects was “invisible” or “theoretical” to the patient (PCP number 5).

#### Patient Misinformation

Clinicians reported patient misinformation as a challenge, describing that some of their patients fall into “misinformation pathways” on the internet, social media, and alternative wellness spaces. Furthermore, clinicians reported difficulty in de-escalating thyroid hormone dose in patients who sought “bioidentical supplements” (PCP number 5) from alternative medicine providers but didn’t disclose such use. Misattribution of general nonspecific symptoms to a thyroid problem was also noted as a barrier to thyroid hormone dose de-escalation.

#### Lack of Familiarity/Trust

The quality of the clinician-patient relationship was reported to be important to successful attempts in de-escalating TSH suppression therapy, particularly for clinicians who “inherited” patients from other clinicians. Clinicians observed that lack of familiarity and of an established relationship with the patient influenced patients’ trust in the decision to de-escalate, especially among those who had a suppressed TSH level for many years. Notably, trust was essential because “anxieties related to a cancer diagnosis” were heightened in the context of thyroid hormone dose reduction (PCP number 1). Participants noted that patients who may not agree with clinicians suggesting thyroid hormone dose de-escalation were likely to seek care elsewhere.

### Clinician-Level Barriers

#### Uncertainty About Thyroid Cancer Risk Stratification

As a barrier to determining goals for TSH suppression, PCPs noted difficulty distinguishing between low- and intermediate-risk thyroid cancer, with one PCP noting “I do not separate these (risk levels) in my mind when a patient comes to me” (PCP number 5). Rather than utilizing the ATA risk stratification guidelines, PCPs reported they assessed risk based on years elapsed since completion of treatment, older age and comorbidities. Although clinicians practicing endocrinology reported using dynamic risk stratification in their patients with thyroid cancer when deciding TSH suppression goals, they reported difficulty making decisions to de-escalate TSH suppression therapy in patients whose TSH fluctuated frequently or fell outside the reference range despite many attempts to adjust the thyroid hormone dose.

#### Variable Use of Guidelines

While clinicians practicing endocrinology reported engagement with the ATA guidelines, they were encumbered by the document’s length, and recognized that few “have time to read several hundred pages” (Endo number 5), especially community clinicians. PCPs were unlikely to proactively utilize the guidelines, in part because their thyroid cancer case volume is low.

#### Unclear Shared Patient Survivorship Goals and Plans Between Specialties

Specialists and PCPs lacked shared clarity about goals, plans, and responsibilities for thyroid cancer survivorship care. PCPs expressed little knowledge or confidence to devise a plan for de-escalating TSH suppression in their patients with recurrence-free, low- or intermediate-risk thyroid cancer. PCPs largely presumed that patients were stable at discharge from endocrinology and that the TSH levels and dosage were on target, particularly in the absence of clear discharge instructions. PCPs found it challenging to become the voice of thyroid hormone dose de-escalation for patients who had not been prepared by their endocrinologists to expect dose reduction after discharge from the specialty clinic. Conversely, without a systematic and consistent hand off from specialty care to PCPs, specialists reported being burdened by ongoing deferral of care from PCPs, and indicated that sometimes patients and PCPs continue to redirect questions about routine surveillance to them, contributing to a backlog of otherwise “graduated” thyroid cancer survivors (Endo number 2).

### System-Level Barriers

#### Clinic Visit Time Constraints

Clinicians practicing in endocrinology indicated that frequent appointments to manage de-escalation, especially with patients who were anxious, contributed to a backlog, such that patients with newly diagnosed thyroid cancer faced longer waiting times for initial visits. Pressure to process their backlog was notable among endocrinologists practicing in large, rural catchment areas with limited specialist care, and for those practicing in denser urban areas that received a high volume of referrals. PCPs reported “limited time and bandwidth” to engage in thyroid hormone dose de-escalation discussions or deprescribing protocols (PCP number 1), especially if it was unrelated to the purpose of the visit nor an issue raised by the patient.

#### Facilitators to De-escalating TSH Suppression

**Fig. 2.**
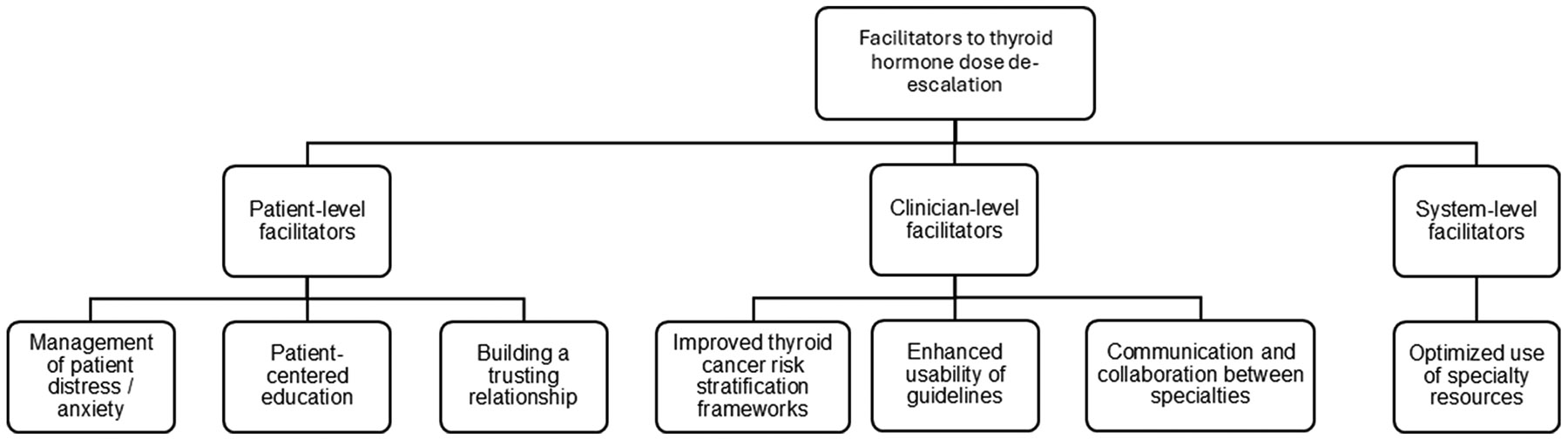
Facilitators to de-escalating thyroid hormone dose in patients with thyroid cancer. Figure 2 shows the emergent themes regarding clinician-reported facilitators to de-escalating TSH suppression therapy in patients with recurrence-free, low- or intermediate-risk thyroid cancer at the patient, clinician, and system levels. *TSH* = thyroid stimulating hormone.

Figure 2 shows the clinician-reported facilitators to de-escalating thyroid hormone dose in patients with recurrence-free, low- and intermediate-risk thyroid cancer. Table 3 displays representative quotations of the themes and subthemes and how they map onto the relevant TDF domains.

### Patient-Level Facilitators

#### Management of Patient Distress/Anxiety

Clinicians discussed practices for managing patient distress and anxiety which centered around rapport with the patient. Participants indicated that when discussing thyroid hormone dose de-escalation, they introduce this topic slowly and reduce the dosage incrementally and gradually. Clinicians reported that treating de-escalation as a “journey” (Endo number 7) and attending to patients’ fears and concerns along the way helps to minimize patient anxiety. The importance of setting expectations for TSH goals and thyroid hormone dose de-escalation early was noted, to ensure patients are not blindsided when the conversation occurs. One participant stated that “de-escalating” may be more readily accepted if framed as “appropriately reassessing” (Endo number 5).

#### Patient-Centered Education

Delivering accurate and up-to-date scientific information in “patient-centered language” (Endo number 4) was a priority to facilitate thyroid hormone dose de-escalation. When educating patients who have been on long-term TSH suppression, an emphasis on “de-programming” previous assumptions and “adapting” to new findings was central (Endo number 8). Participants discussed “getting buy-in” from patients by characterizing novel research findings as “being on the forefront with new and cool data” (Endo number 5). Clinicians reported that sharing their concerns about long-term adverse effects of unnecessary TSH suppression therapy helped to motivate their patients to embrace the “de-escalation journey” (Endo number 7). Finally, participants identified the importance of providing patients with accurate educational materials and resources from societies and patient support organizations.

#### Building a Trusting Relationship

Participants recognized that trusting relationships are important and are easier to cultivate among new patients with whom they can “set the tone” from the outset, as opposed to patients they “inherited” from other clinicians (Endo number 8). Nevertheless, they emphasized that applying shared decision-making bolsters patients’ comfort with “own[ing] their decision” to de-escalate (Endo number 8). Clinicians recognized that saying “No” to patients when warranted is reflective of a “therapeutic relationship” (Endo number 5) and acknowledged that some patients may seek care elsewhere.

### Clinician-Level Facilitators

#### Improved Thyroid Cancer Risk Stratification Frameworks

PCPs expressed strong interest in being guided to apply thyroid cancer risk stratification approaches in their decision-making. Specialists discussed focusing on patient response to treatment rather than “initial risk or recurrence category” (Endo number 4) to make dynamic decisions about de-escalation of TSH suppression therapy.

#### Enhanced Usability of Guidelines

Clinicians practicing in endocrinology reported proactive engagement with guidelines and eagerness to review the 2025 ATA thyroid cancer guidelines, stating there was a general consistency across TSH suppression de-escalating practices within large academic medical centers. PCPs expressed strong interest for an accessible set of guidelines that would be easily operationalized in their clinical contexts. Clinicians indicated higher usability of guidelines if “tools” were embedded within the electronic medical record (PCP number 6), including flags and SmartSets, together with apps or other algorithmic mechanisms. All participating clinicians were enthusiastic about participating in future interventions that would incorporate guidelines into clinical practice in a dynamic and user-friendly manner.

#### Communication and Collaboration Between Specialties

Streamlining communication and collaboration between specialties was identified as a key facilitator to de-escalating TSH suppression therapy. PCPs reported that they “relied” on chart notes to gauge TSH targets for their patients with thyroid cancer (PCP number 7), and they appreciated when treating endocrinologists conveyed a discharge plan for survivorship care. In the absence of such communication, PCPs indicated they would consult an endocrinologist at their institution. PCPs noted that having endocrinologists’ instructions bolstered their “confidence” to de-escalate and the “patients’ trust” in the process (PCP number 3).

### System-Level Facilitators

#### Optimized Use of Specialty Resources

Clinicians reported that more efficient use of time and resources is a key facilitator and identified discharge from the endocrine clinic as a critical inflection point to enhance efficiency. PCPs expressed eagerness for a proactive and informed handoff so they could “take the ball from there” (PCP number 4). Clinicians from endocrinology were mindful to discharge patients from the specialty clinic at optimal timing, noting that if they involved PCPs too soon a “clean break” would be difficult because PCPs and patients would continue to defer thyroid cancer survivorship care to them (Endo number 2).

## Discussion

This novel qualitative study provides important and timely insights into barriers and facilitators to de-escalating TSH suppression therapy in patients with recurrence-free, low- or intermediate-risk thyroid cancer faced by clinicians, identified at the clinician-, patient- and system-levels. Key clinician-reported barriers included patient distress/anxiety and misinformation, unclear shared patient survivorship goals and plans between specialties, and clinic visit time constraints. Clinician-reported facilitators included building a trusting relationship, delivery of patient-centered education, and communication and collaboration between specialties.

Prior studies on de-escalation or deprescribing of medications have focused on older adults with polypharmacy, with the diagnosis of cancer introducing another level of complexity, particularly in advanced cases.^26-31^ Unique from prior studies on de-escalating care, many patients with thyroid cancer have an excellent prognosis, and de-escalation of therapy may reduce patient harm from adverse effects of overtreatment.

Patient-level factors including patient distress and anxiety, and patient misinformation emerged as important barriers to de-escalation of care efforts. Cancer-related-worry is common, including in thyroid cancer survivors with favorable prognosis.^32,33^ Patient fear and anxiety regarding potential side effects from de-escalation of medications has previously been shown as a barrier to deprescribing efforts, particularly due to concern for potential unfavorable outcomes related to return of symptoms or poor outcomes (eg, cancer recurrence).^34-37^ Furthermore, it has been shown that patient misinformation may negatively affect health behaviors.^38,39^ A prior study focusing on older adults without thyroid cancer similarly noted that patient misinformation regarding thyroid hormone medications was a barrier to de-escalation of therapy, suggesting that patient education in addition to anxiety management could be targeted in future interventions.^28^

A key barrier to de-escalation of TSH suppression therapy in patients with thyroid cancer in the current study was the variable use of guidelines due to lack of time or access, or uncertainty regarding how risk stratification schemes translate to patient care. Several tools, such as clinician decision support tools, algorithms or user-centric prompts and alerts, particularly if integrated in the electronic medical record, would enhance usability and consistent use of guidelines. Strategic dissemination of recent guidelines addressing de-escalation of care,^2,9^ not just to endocrinologists, but also to community clinicians, primary care providers (PCPs) and patients, will be key to streamline thyroid cancer survivorship care and cultivate patient buy-in.

Similar to prior studies on de-escalation of inappropriate care, we found that both effective communication and building trusting relationships between clinicians and patients, as well as between PCPs and specialists, are vital to thyroid hormone dose de-escalation in patients with thyroid cancer.^28,40^ Improved workflow and communication, particularly in transitioning from specialists to primary care, and incorporation of risk-benefit communication tools through the electronic medical records would improve thyroid cancer survivorship care.

Our study has several strengths. First, it provides granular information on barriers and facilitators to de-escalation of TSH suppression therapy in patients with thyroid cancer. Second, we used a validated theoretical framework to identify key factors driving behavior change in the context of (de)implementation, enhancing the rigor of our research.^41^ Third, we included both specialists and PCPs, representing different geographical regions and clinical settings, making our findings more generalizable.

Limitations include a small sample and possible selection bias. Studies with narrowly defined objectives, such as this one, show that theoretical saturation can be achieved within 4 focus groups, meaning the point in data collection has been reached where all important issues or insights are exhausted from the data and the emerging themes are comprehensive and well-grounded in the evidence.^22^ While our use of convenience sampling presents risk of selection bias, this method is appropriate for use in this exploratory study.^42,43^ Future large-scale studies that use nonprobability sampling are needed to generate findings that are generalizable to the broader clinician population, including better understanding differences in the de-escalation approach between PCPs and specialists. We acknowledge that our findings may be confounded by the participants’ subjective reporting, which is a limitation inherent to qualitative research. Finally, we recognize that patients also play a crucial role in the decision-making in de-escalating inappropriate TSH suppression in thyroid cancer survivors and future research should also focus on better understanding their preferences and perspectives.

## Conclusion

Our study highlights barriers and facilitators to de-escalating TSH suppression therapy in thyroid cancer survivors at multiple levels, suggesting that future interventions should engage and consider the perspectives of multiple stakeholders. Despite a recent emphasis in de-escalating inappropriate thyroid cancer care,^19,44^ limited data are available in how to implement this in clinical practice. Potential targets for interventions include clinician and patient education, enhanced usability and dissemination of guidelines, and effectively streamlining workflow. De-escalating inappropriate TSH suppression therapy in thyroid cancer survivors can reduce unnecessary patient harm and clinician burden, decrease healthcare resource use and expenditures, and tailor care to disease severity.

## Figures and Tables

**Table 1:** Participant Demographic Characteristics

Characteristics	N (%)
Specialty	
Endocrinology	8 (53)
Primary care (Internal medicine or family practice)	7 (47)
Sex	
Female	11 (73)
Male	3 (20)
Prefer not to say	1 (7)
Practice setting	
Academic	10 (67)
Nonacademic	5 (33)
Years in clinical practice	
0-10	5 (33)
11-20	7 (47)
>20	3 (20)
Geographic region	
Midwest	7 (47)
Northeast	5 (33)
Southwest	2 (13)
Southeast	1 (7)
Practice community	
Suburban	8 (53)
Urban	7 (47)
Thyroid cancer patient volume per mo (mean, range)	
Endocrinology	43 (10-100)
Primary care	1 (1-1)

**Table 2:** Representative Quotes for Barriers to De-escalating Thyroid Hormone Dose in Patients With Thyroid Cancer

Theme	Subtheme	Relevant TDF Domain	Example quotes
Patient-level barriers	Patient distress/anxiety	Emotion	“There's a lot of anxiety – real or perceived – about those things [weight gain hair, loss, general fatigue]. I think that's the biggest barrier for patients.” (Endo #6) “I’ll plan on scheduling a follow up in 3 or 4 mo because I know [my patient] will be calling me and be anxious about the numbers starting to shift.” (Endo #1) “There’s a real dig in the heels [because] the fear of cancer is so high. So, people will cling to that [TSH suppression] to protect cancer [from recurring].” (PCP #5) “There is a lot of anxiety because – you've had thyroid cancer, you had treatment, you don't want it to come back. And so, when there's a lot of anxiety surrounding that, that tends to be when there's more resistance to change.” (PCP #2).
	Patient misinformation	Knowledge	“…the [social media] groups that my patients will get into say, ‘No, I keep hearing about the doctors trying to de-escalate, and everyone says don't do it’.” (Endo #3) People tend to fixate [and] feel that…certain thyroid brands correlate with how they're feeling – their hair, their skin, their weight, their sleep, their energy. (PCP #1) “I think everybody just assumes that when they're fatigued it means they need more thyroid hormone, and I tell patients that their fatigue may very well relate to their insomnia, which is subsequent to their overtreatment. Sometimes that escapes patients.” (Endo #7)
	Lack of familiarity/trust	Socia1 influences	“It's hard when it's a first visit. Especially when it comes from someone older that retired and has the patient for 20, 30 y and you’re the new kid on the block.” (Endo #3) “They may successfully doctor shop with different folks until they find someone who will keep giving them what they want, even though that's to their own detriment.” (Endo #8) “The worst is when they've been treated by somebody for a really long time and now, you're going against that… It's like suddenly, like, who do you trust? (PCP #5).
Clinician-level barriers	Uncertainty about thyroid cancer risk stratification	Knowledge	“I feel like the younger they are, the closer to the cancer diagnosis they are, would make me more hesitant; versus the older they are, the longer it's been, the more comorbidities they have, I would feel better about decreasing it.” (PCP #1) “It's the people who – the Tg [thyroglobulin] just kind of hangs out in that indeterminate range, and you have an intermediate risk at baseline. Those are the ones that you feel like, ‘I should probably run you into the “lower edge of normal territory’… I hold on for a little bit longer before sending them off to their PCP” (Endo #5)
	Variable use of guidelines	Knowledge/skills	“I think there's a lot more consistency and similar practice patterns, because following guidelines – it kind of runs in within our institution. I think the harder thing is the populations that are not part of academic centers.” (Endo #7) “No one has the time to read this [whole thing], especially the community physicians.” (Endo #3). “My impression is most of the people practicing [in the community] who are over suppressing – it isn't because they think that's what they need to do…it's mostly lack of awareness, And that’s [an] issue with guideline implementation – because people haven't heard about it [de-escalation]. I think that's the bigger problem than the willing, ongoing suppression because ‘I disagree with the guidelines.’” (Endo #4). “I would say low confidence [about de-escalating] because I'm not too familiar with guidelines.” (PCP #1).
	Unclear shared patient survivorship goals and plans between specialties	Professional role and identity	“I have low confidence in my knowledge about TSH and what to target for someone who's a thyroid cancer patient and what levels I should be aiming for, if and for how long they should be on treatment.” (PCP #3) “When specialists discharge patients, I think the expectation from primary care is that things are stable. There's no flux, there's no active treatment, there's no active weaning. The patient is stable…My expectation as a PCP would be [for] the specialist discharging, [to…] say, ‘We're discharging. This is what you should be doing long term.’ (PCP #6)
System-level barriers	Clinic visit time constraints	Environmental context and resources	“My next available [appointment] is like 6 plus mo from now.” (Endo #5) “When we're really busy in clinic it's just not going to pass our minds” (PCP #6) “Given all the other things we have to do, [PCPs] would not touch this…unless the patient brought it up (PCP #5).”

Abbreviations: PCP = primary care provider; TDF = Theoretical Domains Framework.

**Table 3:** Representative Quotes for Facilitators to De-escalating Thyroid Hormone Dose in Patients With Thyroid Cancer

Theme	Subtheme	Relevant TDF Domain	Example quotes
Patient-level facilitators	Management of patient distress/anxiety	Emotion	I usually don't try to jump too fast. I'm like, ‘Oh, let's try just a little bit; just half a tablet,’ and you kind of convince them, little by little, that low TSH was not needed…So, I think it's just *a lot* of conversation, but you can't jump too fast. If you jump too fast you lose the patient.” (Endo #3) “I work on reducing the dose gradually. Kind of meeting her and addressing her fears and taking it slowly in that de-escalation. Going on the journey, rather than just abruptly drop it to some dosage of your choosing.” (Endo #7) “If we've already had the conversation that – as time goes on and the years go on, if there's no sign of recurrence [then] we're going to back off on the dose – when it comes time to do that they're less anxious about that, because they already knew. I think involving them in in the ‘Why’ and the ‘How’ of their disease is helpful in getting good compliance.” (Endo #2) “You can't just have anyone deliver this. It can't be a sub in. That would not be the place to have this discussion. I think also that this needs to be prepped, and a continuous consistent message from the Endo team all the way through to us [PCPs].” (PCP #5)
	Patient-centered education	Knowledge	“I would share with this patient that there's been a bit of a conflict in the literature about whether you need a TSH target after hemithyroidectomy, and most of the studies are retrospective. I would put that in patient-centered language.” (Endo #4) “I sometimes take advantage [and say], ‘Look at all these cool studies and look, things have changed in the last 10 y’ And, ‘Have you heard about this revolution in medicine and all these really cool studies that have come out since? They're super cool, they changed everything.’ And sometimes I get buy in from that when they're like, ‘I want to be on the forefront of new and sexy cool data and medicine.’” (Endo #5) “Calling it de-escalating is a much harder sell to patients than [saying], ‘We're appropriately reassessing based on the fact that patients do amazing’ … There’s a significant discrepancy between how we’re framing it versus how patients feel about it.” (Endo #5)
	Building a trusting relationship	Social influences	“It may take some patients several visits. It may take some time until you have that relationship, that trust. I think, over time, most of them get it.“ (Endo #8) “Really, truly, shared decision making helps a lot. I usually do shared decision making with people, sharing that uncertainty about ‘We don't think it raises risk for recurrence’… I've had a pretty good experience with people [then] wanting to [de-escalate].” (Endo #4) “[With shared decision making], the patients know they own their decision. So, it's not somehow me telling them what to do, but it's them making that decision in conjunction with their provider and say[ing] ‘This is what we need to do together as one team.’ And most of the time, they’ll feel comfortable, knowing that was their decision.” (Endo #8)
Clinician-level facilitators	Improved thyroid cancer risk stratification frameworks	Skills	“One thing about primary care physicians, we're super scrappy, and we will step in and do stuff…I think we just need to be given the tools. And then a little bit of training.” (PCP #5) “I find the dynamic risk re-stratification is such an important part of this. We're talking about lower intermediate risk, but I'm usually not focusing on what their initial risk or recurrence category was the further you go out from the cancer. For me, it really comes down to, ‘Are they in the excellent response, category? Indeterminate? Or are they biochemically incomplete or structurally incomplete?’” (Endo #4)
	Enhanced usability of guidelines	Behavioral regulation	“I wouldn't stop it [thyroid hormone] on my own because I've just not been guided through it. If there was a guideline for it, I for sure would do it. I would love to do it.” (PCP #5) “I think if I just had a plan written out from the endocrinologist about, ‘This is what for this patient I would recommend.’ That would give me a lot of confidence in managing them in the future.“ (PCP #1) “I think that creating some sort of an algorithmic tool will definitely be much more help. And it can be even an app on the phone, or something [to] make it easier for the community physicians. They are the ones that probably would benefit the most. (Endo #3)
	Communication and collaboration between specialties	Social influences/professional role and identity	“I do rely heavily on endocrine. I will look back at the note to see like what their TSH suppression goal is. I just rely on endocrine for that.” (PCP #7) “I do find that patients don't necessarily trust me, but if I say, ‘Okay, I talked to the endocrinologist, and this is what they're recommending’.” (PCP #7) “Most of the time they [patients] are still seeing a thyroid specialist until 5 y and then the thyroid specialist will say, ‘These are your goals, and this is what you should be doing.’ And I like it when they do it because then the patients that are like, ‘That's what my thyroid doctor said.’” (PCP #2) “Some of my patients are fine with what I say. And then some of them are like, ‘Well, you aren't the specialist.’ So being able to [say], ‘Hey, this is coming from the team, and from your specialist who is the one who put forth the plan’ really helps give us confidence to have the conversation.“ (PCP #3)
System-level facilitators	Optimized use of specialty resources	Environmental context and resources	“In terms of when to involve primary care, if you try to get them involved too early it's just one more thing. [So] it does end up being kind of messy…they'll [patients] still call me with their labs, even though their primary is supposed to be interpreting it. And so, that gets clunky…when we try to involve them. Has to be kind of a clean break, with clean recommendations. Clear guidelines as to when to return.” (Endo #1) “From a systems view, maybe there's shared decision making about who wants to manage it [de-escalation]. For example, in other cancers, there may be a little less of a sharp cut off between transition from oncology to PCP. Sometimes I look and wonder, ‘Why are they going back to their oncologist every year to do the same 2 tests? I could do this.’” (PCP #5).

Abbreviations: PCP = primary care provider; TDF = Theoretical Domains Framework; TSH = thyroid stimulating hormone.

## Highlights

- Current guidelines advocate for a de-escalation of thyroid stimulating hormone (TSH) suppression therapy with a goal serum TSH in the normal reference range in patients with recurrence-free, low- or intermediate-risk thyroid cancer
- Key barriers to TSH suppression de-escalation faced by clinicians include patient distress/anxiety and misinformation, unclear shared patient survivorship goals and plans between specialties, and clinic visit time constraints
- Key facilitators include establishing trust with patients, offering education tailored to patient needs, and promoting interspecialty communication and collaboration to develop unified long-term survivorship plans
- Future interventions towards deimplementation of inappropriate TSH suppression therapy in thyroid cancer survivors should incorporate input and perspectives of multiple stakeholders

## Clinical Relevance

Understanding barriers and facilitators to de-escalating thyroid stimulating hormone suppression therapy in survivors with recurrence-free, low- or intermediate-risk thyroid cancer is vital to optimizing survivorship care and reducing patient harm. These study results represent an initial step toward minimizing unnecessary thyroid stimulating hormone suppression among thyroid cancer survivors.

## Abbreviations:

ATA: American Thyroid Association
PCP: primary care provider
TDF: Theoretical Domains Framework
TSH: thyroid stimulating hormone

## References

[1] NIH National Cancer Institute. Surveillance, epidemiology, and end results program. Cancer Stat Facts: Thyroid Cancer. https://seer.cancer.gov/statfacts/html/thyro.html. Accessed June 11, 2025.

[2] Ringel MD, Sosa JA, Baloch Z, 2025 American thyroid association management guidelines for adult patients with differentiated thyroid cancer. Thyroid. 2025;35(8):841–985. 40844370 10.1177/10507256251363120 PMC13090833

[3] Haugen BR, Alexander EK, Bible KC, 2015 American thyroid association management guidelines for adult patients with thyroid nodules and differentiated thyroid cancer: the American thyroid association guidelines task force on thyroid nodules and differentiated thyroid cancer. Thyroid. 2016;26(1):1–133. 26462967 10.1089/thy.2015.0020 PMC4739132

[4] Biondi B, Cooper DS. Thyroid hormone suppression therapy. Endocrinol Metab Clin North Am. 2019;48(1):227–237. 30717904 10.1016/j.ecl.2018.10.008

[5] Carhill AA, Litofsky DR, Ross DS, Long-term outcomes following therapy in differentiated thyroid carcinoma: NTCTCS registry analysis 1987-2012. J Clin Endocrinol Metab. 2015;100(9):3270–3279. 26171797 10.1210/JC.2015-1346 PMC5393522

[6] Jonklaas J, Sarlis NJ, Litofsky D, Outcomes of patients with differentiated thyroid carcinoma following initial therapy. Thyroid. 2006;16(12):1229–1242. 17199433 10.1089/thy.2006.16.1229

[7] Qiang JK, Sutradhar R, Everett K, Association between serum thyrotropin and cancer recurrence in differentiated thyroid cancer: a population-based retrospective cohort study. Thyroid. 2025;35(2):208–215. 39723994 10.1089/thy.2024.0330

[8] Braafladt S, Allison H, Chung J, Dose-dependent relationship between levothyroxine and health-related quality of life in survivors of differentiated thyroid cancer. Surgery. 2025;179:108799. 39341744 10.1016/j.surg.2024.07.057

[9] NCCN Clinical Practice Guidelines in Oncology (NCCN Guidelines^®^). Thyroid Carcinoma; 2025. Version 1.2025.

[10] Papaleontiou M, Chen DW, Banerjee M, Thyrotropin suppression for papillary thyroid cancer: a physician survey study. Thyroid. 2021;31(9):1383–1390. 33779292 10.1089/thy.2021.0033 PMC8558057

[11] Shi X, Tang H, Zhang T, Thyroid-stimulating hormone suppression in low-risk papillary thyroid cancer: a large-scale retrospective analysis of real-world data. EClinicalMedicine. 2024;77:102912. 39534024 10.1016/j.eclinm.2024.102912 PMC11555599

[12] Evron JM, Hummel SL, Reyes-Gastelum D, Association of thyroid hormone treatment intensity with cardiovascular mortality among US veterans. JAMA Netw Open. 2022;5(5):e2211863. 35552725 10.1001/jamanetworkopen.2022.11863 PMC9099430

[13] Papaleontiou M, Levine DA, Reyes-Gastelum D, Thyroid hormone therapy and incident stroke. J Clin Endocrinol Metab. 2021;106(10):e3890–e3900. 34137866 10.1210/clinem/dgab444 PMC8475197

[14] Park J, Blackburn BE, Ganz PA, Risk factors for cardiovascular disease among thyroid cancer survivors: findings from the Utah cancer survivors study. J Clin Endocrinol Metab. 2018;103(7):2468–2477. 29850817 10.1210/jc.2017-02629 PMC6915829

[15] Papaleontiou M, Banerjee M, Reyes-Gastelum D, Risk of osteoporosis and fractures in patients with thyroid cancer: a case-control study in U.S. veterans. Oncologist. 2019;24(9):1166–1173. 31164453 10.1634/theoncologist.2019-0234 PMC6738319

[16] Wang LY, Smith AW, Palmer FL, Thyrotropin suppression increases the risk of osteoporosis without decreasing recurrence in ATA low- and intermediate-risk patients with differentiated thyroid carcinoma. Thyroid. 2015;25(3):300–307. 25386760 10.1089/thy.2014.0287 PMC6916125

[17] Shah K, Reyes-Gastelum D, Gay BL, Understanding worry about risks associated with thyroid hormone therapy: a National survey of endocrinologists, family physicians, and geriatricians. Endocr Pract. 2021;28(1):25–29. 34438052 10.1016/j.eprac.2021.08.007 PMC8748409

[18] Hughes DT, Reyes-Gastelum D, Kovatch KJ, Energy level and fatigue after surgery for thyroid cancer: a population-based study of patient-reported outcomes. Surgery. 2020;167(1):102–109. 31582311 10.1016/j.surg.2019.04.068 PMC6904434

[19] Haymart MR, Goldner WS. Thyroid cancer clinical guidelines and the De-escalation of care. JAMA Otolaryngol Head Neck Surg. 2020;146:1082–1083. 33022039 10.1001/jamaoto.2020.3260

[20] Enhancing the QUAlity and Transparency Of health Research. Standards for reporting qualitative research: a synthesis of recommendations. https://www.equator-network.org/reporting-guidelines/srqr/; 2023. Accessed July 2, 2025.

[21] Palinkas LA, Horwitz SM, Green CA, Purposeful sampling for qualitative data collection and analysis in mixed method implementation research. Adm Policy Ment Health. 2015;42(5):533–544. 24193818 10.1007/s10488-013-0528-y PMC4012002

[22] Hennink M, Kaiser BN. Sample sizes for saturation in qualitative research: a systematic review of empirical tests. Soc Sci Med. 2022;292:114523. 34785096 10.1016/j.socscimed.2021.114523

[23] Guest G, Namey E, McKenna K. How many focus groups are enough? Building an evidence base for nonprobability sample sizes. Field Methods. 2017;29(1):3–22.

[24] Atkins L, Francis J, Islam R, A guide to using the theoretical domains framework of behaviour change to investigate implementation problems. Implement Sci. 2017;12(1):77. 28637486 10.1186/s13012-017-0605-9 PMC5480145

[25] Bingham AJ. From data management to actionable findings: a five-phase process of qualitative data analysis. Int J Qual Methods. 2023;22:16094069231183620.

[26] Turner JP, Kantilal K, Kantilal K, Optimising medications for patients with cancer and multimorbidity: the case for deprescribing. Clin Oncol (R Coll Radiol). 2020;32(9):609–617. 32563549 10.1016/j.clon.2020.05.015

[27] Linsky AM, Motala A, Booth M, Deprescribing in community-dwelling older adults: a systematic review and meta-analysis. JAMA Netw Open. 2025;8(5):e259375. 40338546 10.1001/jamanetworkopen.2025.9375 PMC12062908

[28] Moretti B, Livecchi R, Taylor SR, Physician-reported barriers and facilitators to thyroid hormone deprescribing in older adults. J Am Geriatr Soc. 2025;73(2):566–573. 39392046 10.1111/jgs.19219 PMC11828684

[29] Martin P, Tamblyn R, Benedetti A, Effect of a pharmacist-led educational intervention on inappropriate medication prescriptions in older adults: the D-PRESCRIBE randomized clinical trial. JAMA. 2018;320(18):1889–1898. 30422193 10.1001/jama.2018.16131 PMC6248132

[30] Burgos N, Toloza FJK, Singh Ospina NM, Clinical outcomes after discontinuation of thyroid hormone replacement: a systematic review and meta-analysis. Thyroid. 2021;31(5):740–751. 33161885 10.1089/thy.2020.0679 PMC8110016

[31] Maraka S, Owen RR, Singh Ospina NM, Discontinuation of levothyroxine therapy in patients with subclinical hypothyroidism: a pilot randomized clinical trial. Endocrine. 2025;90(2):781–792. 40736623 10.1007/s12020-025-04371-z PMC12364413

[32] Papaleontiou M, Zebrack B, Reyes-Gastelum D, Physician management of thyroid cancer patients’ worry. J Cancer Survivorship. 2020;15(3):418–426. 10.1007/s11764-020-00937-0 PMC7960572 32939685

[33] Papaleontiou M, Reyes-Gastelum D, Gay BL, Worry in thyroid cancer survivors with a favorable prognosis. Thyroid. 2019;29(8):1080–1088. 31232194 10.1089/thy.2019.0163 PMC6707035

[34] Gillespie RJ, Harrison L, Mullan J. Deprescribing medications for older adults in the primary care context: a mixed studies review. Health Sci Rep. 2018;1(7):e45. 30623083 10.1002/hsr2.45 PMC6266366

[35] Sinnott C, Hugh SM, Boyce MB, What to give the patient who has everything? A qualitative study of prescribing for multimorbidity in primary care. Br J Gen Pract. 2015;65(632):e184–e191. 25733440 10.3399/bjgp15X684001 PMC4337307

[36] Wallis KA, Andrews A, Henderson M. Swimming against the tide: primary care physicians' views on deprescribing in everyday practice. Ann Fam Med. 2017;15(4):341–346. 28694270 10.1370/afm.2094 PMC5505453

[37] Reeve E, To J, Hendrix I, Patient barriers to and enablers of deprescribing: a systematic review. Drugs Aging. 2013;30(10):793–807. 23912674 10.1007/s40266-013-0106-8

[38] Borges do Nascimento IJ, Pizarro AB, Almeida JM, Infodemics and health misinformation: a systematic review of reviews. Bull World Health Organ. 2022;100(9):544–561. 36062247 10.2471/BLT.21.287654 PMC9421549

[39] The Physicians Foundation. The effect of misinformation and disinformation on physicians’ ability to provide quality care. https://physiciansfoundation.org/research/the-effect-of-misinformation-and-disinformation-on-physicians-ability-to-provide-quality-care/; 2025. Accessed August 26, 2025.

[40] Jansen J, Naganathan V, Carter SM, Too much medicine in older people? Deprescribing through shared decision making. BMJ. 2016;353:i2893. 27260319 10.1136/bmj.i2893

[41] Johnson JL, Adkins D, Chauvin S. A review of the quality indicators of rigor in qualitative research. Am J Pharm Educ. 2020;84(1):7120. 32292186 10.5688/ajpe7120 PMC7055404

[42] Ahmed SK. How to choose a sampling technique and determine sample size for research: a simplified guide for researchers. Oral Oncol Rep. 2024;12(100662):1–7.

[43] Emerson RW. Convenience sampling revisited: embracing its limitations through thoughtful study design. J Vis Impairment Blindness. 2021;115(1):76–77.

[44] Perkins JM, Papaleontiou M. Towards De-Implementation of low-value thyroid care in older adults. Curr Opin Endocrinol Diabetes Obes. 2022;29(5):483–491. 35869743 10.1097/MED.0000000000000758 PMC9458619

